# Promoting Self-Care in Nursing Encounters with Persons Affected by Long-Term Conditions—A Proposed Model to Guide Clinical Care

**DOI:** 10.3390/ijerph18052223

**Published:** 2021-02-24

**Authors:** Carina Hellqvist

**Affiliations:** 1 Department of Neurology, University Hospital Linköping, 58185 Linköping, Sweden; carhe89@liu.se; 2 Department of Health, Medicine and Caring Sciences, Linköping University, 58183 Linköping, Sweden

**Keywords:** self-care, self-management, nursing support, long-term condition, Parkinson’s disease

## Abstract

Background: Nursing interventions for persons affected by long-term conditions should focus on providing support to enhance the ability to manage disease in everyday life. Many clinical nurses feel they have inadequate training or experience to provide self-management support in a beneficial and structured way. This study explores the process towards independent self-care and management of disease in persons affected by Parkinson’s disease and the support required from healthcare to achieve this. It presents a nursing model to guide nurses in providing self-management support in the clinical care encounter. Methods: The results from three previously published articles investigating a self-management support program for persons with Parkinson’s disease were combined to form a new data set, and analyzed using qualitative thematic analysis. Results: Three separate, but interrelated, themes were identified, which described the process towards self-management of disease as expressed by the participants of the self-management program. Themes describe the factors important for developing and improving self-management abilities and actions. The results were applied to Orem’s Self-care deficit theory to suggest a model of self-management support in the clinical nursing encounter. Conclusion: This study investigated factors important for self-management and highlighted the unique contribution and focus of nursing support to promote independent self-care.

## 1. Introduction

Long-term conditions, sometimes referred to as non-communicable diseases, largely affect the resources available in healthcare and are responsible for approximately 70% of all deaths worldwide [1]. A longer life expectancy is rapidly changing population demographics leading to a larger number of persons above the age of 60 years. With increasing age, the likelihood of being affected by a long-term condition is also higher. These conditions often require major adjustments and adaptations in life for the person affected, and recurrent and regular monitoring and contacts with healthcare services [2]. Persons with long-term conditions are encouraged and expected to be active participants in their own care and well-being. Skills and strategies to handle the impact of disease in everyday life are vital for persons to undertake adequate self-care actions, and thereby, maintain a sense of control, to manage symptoms and maintain satisfaction in their lives. For nurses working in outpatient settings, the provision of self-management support to persons with long-term conditions is considered a major part of professional care [3]. Nursing care is considered holistic in nature, focusing on the entire life-situation and wellbeing of the person, not only the medical aspects of disease [4]. Nurses are recognized as having the appropriate medical knowledge, and are often easily available for persons seeking guidance and support on how to handle the impact of disease on everyday life. These pre-requisites place nurses in the optimal position to provide self-management support. Nevertheless, many nurses feel uncertain about how to provide self-management support and do not feel that they have received sufficient education and training in skills needed to provide this [3,5].

Strengthening patients’ abilities for self-care actions is the main goal of nursing care according to Dorothea Orem´s Self-care deficit theory. This general and well-known theory of nursing is based on the assumption that all nursing interventions should be aimed at strengthening patients’ own ability to perform self-management activities to manage their own self-care “in order to maintain life, health and well-being” [6]. Orem acknowledges self-care to be a broad concept concerning all aspects of life and not just management of disease, although nursing support primarily focuses on support of self-care deficits due to the impact of disease or ill health. The self-care deficit theory is partly concerned with the more philosophical underlying assumptions of nursing, but above all, it is an action-theory intended to be used in clinical work. The theory has clear specifications for the nurse and patient roles in order to enhance self-care [6,7].

Parkinson´s disease (PD) is an example of a long-term condition primarily present in the older population. PS is a slowly progressing neurological condition diagnosed in approximately 1% of persons above the age of 60 years, meaning that about 6.1 million persons in the world are living with this long-term condition [8]. In PD the loss of dopaminergic neurons in the brain lead to symptoms affecting movement and tremors as well as psychiatric symptoms like anxiety and depression. Although good medical treatment is available to manage symptoms, it will not cure or halt disease progression [9]. Cognitive decline and dementia are common in the later stages of disease. Persons with PD will live for decades with slowly progressing symptoms increasingly affecting everyday life for the person, as well as for close family members and spouses. Persons with PD are primarily cared for in outpatient clinics and supported by multidisciplinary teams to monitor symptoms, adjust treatment, and provide practical and emotional support [10]. Admissions to hospitals are rare. Specialized nurses, often called Parkinson’s Disease Nurse Specialists (PDNS), work in outpatient care to support persons with PD in many countries across the world. Guidelines describing the PDNS work have been developed in several countries, and the PDNS function is considered an important part of providing high-quality care for this group of patients [11,12,13]. Nevertheless, even with the regular support of a multidisciplinary team of specialized healthcare professionals, the management of medication and symptoms in everyday life are the concerns of persons with PD and close family members. To be able to live a good and fulfilling life in spite of PD, both the persons affected, as well as their care partners need good skills in managing and monitoring the disease and they need the ability to perform self-care actions [14,15]. Several self-management support programs, specifically tailored to persons with PD, are provided and tested in clinical care. Most of these programs are designed as small group interventions where participants meet in person for a number of sessions and provided on site at the outpatient clinic. Although the programs have been evaluated scientifically, many studies are using small samples recruiting participants from only one study-site and included different outcome measures. The contents of the programs also show substantial diversity, some focusing more on provision of medical information, whilst others include one or more strategies for problem-solving, action planning, self-monitoring and behavioural change. A majority of studies also adopted a research design that is not suitable for comparisons and the effectiveness and outcomes. Although self-management support is provided to patients with PD in clinical care, there are still large knowledge gaps concerning what these interventions should include in terms of their contents, the best way of delivery, as well as the expected outcomes for participants and for healthcare [16,17]. In recent years, technological advances have provided new opportunities in clinical care [18]. Technical devices are now used for symptom monitoring, initiation and titration of treatments for and provision of therapies for example cognitive behavioural therapy in Parkinson’s disease [19,20]. In the coming years technology will also be more integrated in the way self-management support is carried out. Technical apps and platforms provide alternative ways of self-management support provision for both group and individual interventions [21,22].

Previous studies addressing PDNS function have mentioned self-management support as one important part of a multi-dimensional role together with several other important tasks [11,12]. This study is the first one to explore and describe in depth the contents and interactions present in PDNS self- management support, using a general nursing framework to promote understanding of how this can be performed in the clinical encounter with patient and care partner.

The aim of this study was two-fold. Firstly, to explore self-management of disease as it was expressed by persons with Parkinson´s disease and their care partners when participating in the self-management intervention, the Swedish National Parkinson School. A better understanding of what the persons themselves find important to be able to manage disease in everyday life can provide insight for nurses into the preferred focus of their nursing interventions and guide how these interventions can be performed in clinical care. The second aim was to apply the findings to a general nursing theory of self-care, Dorothea Orem’s self-care deficit theory, to elaborate on how the results can be used in clinical care, as well as how the nursing actions to support self-management can be understood and carried out in the clinical encounter.

## 2. Materials and Methods

This study used qualitative and explorative design with a two-step analysis applying both inductive and deductive approaches. The first inductive step of analysis in this study was conducted as a secondary analysis of data [23]. Three previously published articles were included [24,25,26] (Table 1), all of which investigated the outcomes of a dyadic self-management support program: Swedish National Parkinson School (NPS) for persons with PD and their care partners. The results sections of these articles together formed the new set of data analysed in this study. There are only three studies available investigating the NPS. Therefore, sampling included all three published studies. The secondary analysis was performed in order to move focus from merely evaluating the outcomes of the educational intervention and instead aim to learn about the self-management process, and facilitators of and barriers to self-management from the perspective of the participants. The author was involved in the parent studies and is well familiar with the data collection and research methods that was used to achieve the results in these. In the second step of analysis, in the current study, the results from step one were deductively applied to Dorothea Orem´s Self-care Deficit Nursing theory [6]. This was done to interpret the results within a framework that could be used to guide nurses engaging in self-care support in clinical settings.

### 2.1. Intervention

The Swedish National Parkinson School (NPS) is a dyadic self-management intervention for persons with PD and their care partners. The program was developed in 2013, and has been provided in clinical practice since 2014 in neurologic and geriatric outpatient clinics across Sweden [27,28]. The focus of the NPS is to handle symptoms of Parkinson´s disease in everyday life, by introducing techniques and strategies for self-monitoring, planning ahead, taking action, positive thinking, communication and resource utilisation. Participants of the NPS meet in a small group once a week for total of seven sessions, each session being two hours long. Every session provides information about a topic relevant to everyday life with Parkinson’s disease followed by a group discussion stimulating interaction and peer-support between the participants. Between the sessions, participants have the opportunity to reflect on, and try out, what has been discussed during the NPS in their own everyday lives through home-assignments. The contents of the NPS were influenced by a previous European project that resulted in a model of patient education specifically tailored to this group of patients with PD called PEPP [29]. (For more information about the NPS see supplementary material)

### 2.2. Participants and Settings

In total, 127 persons with PD and 75 care partners from five separate outpatient clinics across Sweden were included in the material used for this study. The three studies included used different perspectives and methodologies to explore the outcomes of the NPS. Study I is a qualitative study with data consisting of audio recordings of the last group session of the NPS educational program, analysed with qualitative thematic analysis [30,31]. Study II is a quantitative quasi-experimental case-control study using self-reported questionnaires administered before and after participation in the NPS program or 7 weeks of standard care. Statistic methods comparing results within and between groups were used to analyse data [32] Study III is a qualitative study with data consisting of observations and follow-up interviews of a clinical encounter and analysed with constant comparative analysis [33,34]. (for an overview of methods and participants see Table 1). All studies were conducted with the permission of the regional ethical review board and participants gave written and verbal consent following the declaration of Helsinki [35].

### 2.3. Data Analysis

#### 2.3.1. Inductive Secondary Analysis of Data

**Figure 1:**
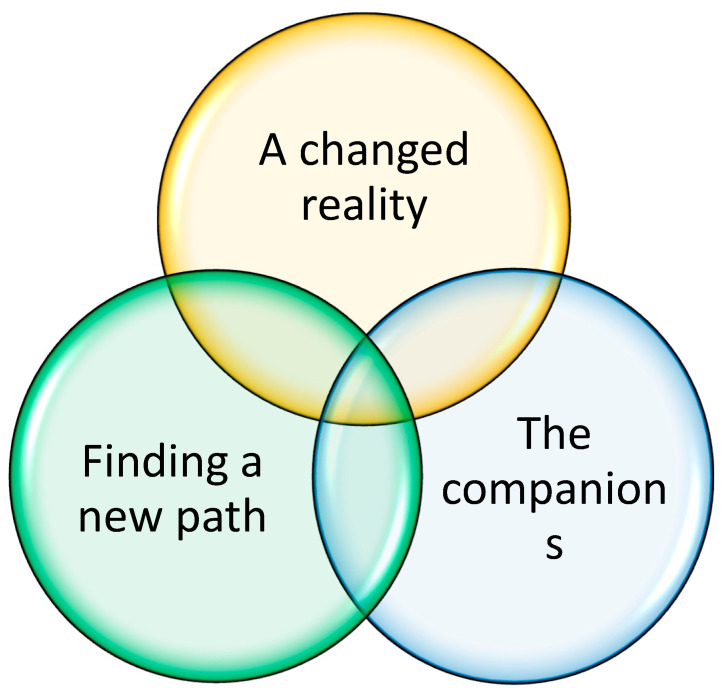
The over-all results of the inductive qualitative thematic analysis. Three interrelated themes describing the building blocks and process towards self-management of PD for persons affected and their care partners as described by participants of the Swedish National Parkinson School.

Qualitative thematic analysis, as described by [23], was chosen for compiling and performing secondary analysis of data. The method is not tied to any specific theory or underlying philosophy, and could be applied to several types of data and data sources. It allows for comparison and analysis of both qualitative and quantitative data together as parts to form a new whole. The method has also previously been used in studies carried out in clinical care settings [31]. An inductive approach to data was used with no previous theory or framework to guide analysis. A realist semantic standpoint was adopted for the analysis to remain close to the data and the actual words and experiences as expressed by the participants in the studies. This was important as the focus of the study was to explore participants’ views. The analysis was performed by the author of this study who is a registered nurse and has a PhD with previous experience of using a qualitative thematic method to analyse data. The author is also working clinically in an outpatient care setting to support persons affected by PD and their care partners. During the first step of the analysis all three studies were read several times to get a sense of the whole body of data. Initial thoughts about the contents were written down. After this, each study was read more thoroughly and codes relevant to the research question were extracted. Codes from all studies were then compared and related codes were sorted into broader themes. Initially six themes were identified but some themes were found to describe the same phenomenon from another angle, and were, thus, incorporated and combined. The final result of this first step using inductive and qualitative analysis consists of three related but separate themes describing the process and activities of self-management, including the need and wishes for self-management support from the perspective of the participants (see Figure 1) The final themes were checked against the initial text to confirm and identify them in the initial text. The 15-step checklist for good quality thematic analysis as provided by Braun and Clarke was followed throughout the analysis to ensure structure and quality [30].

#### 2.3.2. Deductive Application of Results

To explore how the results retrieved in step one could be understood and applied in the clinical practice of nurses working to support and enhance self-management in persons with long-term conditions, the second step of analysis applied the findings to Dorothea Orem’s Self-care deficit nursing theory [6]. The application was undertaken to evaluate if the theory could be used to guide nurses in their work to provide self-management support in clinical care settings. The three themes obtained in the first step of inductive analysis resulted in suggestions for nursing actions appropriate to support patients’ and care partners’ abilities to engage in self-care. These were checked for their fit into the framework of Orem’s Self-care deficit theory. This second part of analysis generated a model describing the nurse/patient/caregiver interaction to support self-care during a clinical encounter and is presented in the results section below. The model obtained through analysis was distributed to other nurses working in clinical care to support persons with PD and care partners to gather their views on the usefulness and applicability in clinical practice.

## 3. Results

### 3.1. Inductive Thematic Analysis

The first inductive step of secondary analysis of the three studies resulted in three distinct but related themes. These themes are describing the process of self-management of disease and the important conditions for enhancing self-management abilities and actions in everyday life, as expressed by persons with PD and their care partners during participation in the Swedish National Parkinson School (NPS). (Figure 1) The contents of each theme is described and supported with quotations from the original studies below.

#### 3.1.1. Theme 1 “A Changed Reality”

Persons with PD and care partners both acknowledged the time of diagnosis as the starting-point of a new era in life, as well as the starting-point in the process towards self-management of PD in everyday life. To be informed of a lifelong chronic illness was experienced by many as a shock and brought about emotional reactions like anger, denial and sadness.

“When you are first diagnosed with the disease you feel very lonely. You have a lot of questions about the future and maybe see the worst scenario in front of you” (Paper III, Participant 1, page 3).

Receiving a diagnosis appeared to change the very foundations of life and also affected relationships in the family and between husbands and wives. Persons affected by PD also felt that their perceptions about themselves, i.e., their personal identity, was altered. Persons with PD, as well as the care partners expressed worries about the future, how rapidly the disease would progress and affect mental and physical abilities and how this would impact life ahead. For many, the initial period after the diagnosis was primarily dominated by strong feelings and emotional reactions and a loss of control over life. The first attempts to accept and manage the changed reality included speaking openly to others about the diagnosis. Also upward and downward social comparison was used to relieve stress in their own life. Statements like “there is always someone worse off”, “ it is not a fatal disease “ or “ there are good medical treatments available now to manage symptoms of disease” were common.

“I still think that I’ve been fortunate. Because there are others that are much worse off than I am. I don’t have any problems walking. I can walk without aids!” (Paper III, Participant 8, page 2).

#### 3.1.2. Theme 2 “Finding a New Path”

After the initial period of strong emotions at diagnosis, most participants found a more balanced way to deal with the new circumstances of life. They started to come to terms with PD as being a part of life and with this new outlook they were planning for life ahead. When participants, both persons with PD and their care partners, reached this mental state of acknowledging the present situation, they were also ready and willing to find new ways to deal with the physical, as well as emotional symptoms of disease. Many felt that acquiring new knowledge about the disease itself, including medical treatments available, was one way to feel more in control of the situation. Also thinking difficult situations through, and make action-plans for how to handle them, was helpful. Learning techniques for self-monitoring symptoms was helpful in managing them when they occurred in everyday life and also helped to alert patients to contact healthcare professionals when new symptoms occurred and to communicate the current health status to care providers. These skills were achieved to some extent by the participants in the NPS program.

“Results of participation in the NPS could be measured as a shift in how persons view the impact of disease in their everyday lives and are connected to a mind-set of not allowing the disease to control life… [This mind-set reflects] better knowledge of skills and techniques to manage and cope with the impact of PD.” (Study II, results page 10)

Trying to keep a positive mindset and to be active participants in their own lives, prioritizing activities that brought feelings of happiness and joy were important to deal with both the physical and emotional impact of PD in daily life.

“I sing in a choir. Singing makes me feel good, even if you felt exhausted before going you feel really good afterward.” (Study III, Participant 6, page 3).

Engaging in physical activities was a common way to maintain physical abilities as well as to enhance emotional well-being. Participants also adopted many self-care strategies to deal with the more practical impact of PD; these included, for example, strategies to facilitate dressing, managing personal hygiene and intake of medication.

#### 3.1.3. Theme 3 “The Companions”

Adjusting to PD clearly involves more people than the person affected by the disease. The new skills and abilities needed to manage PD were developed in a social context including interactions with spouses, other members of the family and friends. Also healthcare professionals were important in providing advice and guidance. These people surrounding the person affected by PD can be viewed as “companions” to provide support and help them on the path of self-management. Their actions most often support and encourage self-care but can occasionally hinder self-care abilities. If persons close to them were perceived as being supportive and tolerant this would ease the psychological strain of being affected by the disease. The person closest is often a spouse. Many spouses expressed a view that managing PD in everyday life is a shared mission, and not just a concern of the person affected by the disease. Based on this view, spouses would also act as care partners and the approach of a joint concern was found to be beneficial, strengthening the relationship and promoting well-being for both. There were several examples of support from spouses in managing PD in everyday life, which included providing motivation and support in physical activities and activities of daily living, support in following medication regimens and accompanying patients at medical appointments. The amount and type of support provided by the care partner differed according to the progression of the disease. Many care partners expressed a wish for healthcare to teach them methods and strategies to provide further support for their partner in managing several aspects of the disease in order to maintain healthy behaviours and physical and psychological well-being. The NPS provided an opportunity to meet others in the same situation. This was found to be valuable and it was much appreciated by the participants in discussing with others how to handle situations in life brought on by PD and to give each other advice on how to handle these instances made them feel less isolated and lonely in their life situation.

“My feeling is that this group has been a helping hand because it has been very difficult trying to carry it all by myself… It’s been a lifesaver… I feel that my husband and I have a future to look forward to” (Study I, Group 1 care partner, row 1110–1115).

Even though the social surroundings, and support offered by the persons close by and available in everyday life, undoubtedly were most important for persons with PD and care partners, the participants also expressed that the support given by health care professionals was important in guiding them towards self-management. Persons with PD would seek healthcare professionals for general information and support concerning the disease itself including pathophysiology, symptoms, progression and medical treatments. They would also seek advice on how to handle specific symptoms of the disease, i.e., obstipation or hallucinations when they occurred in everyday life as a result of PD. Healthcare professionals were also involved in providing knowledge of strategies to monitor symptoms and to grasp which signs could indicate progression of disease, and to explain important information to communicate in encounters with healthcare to adjust care and medical treatment; they also wanted information about which symptoms or situations should trigger persons with PD, and care partners themselves to actively seek medical help if they occurred in everyday life. A good relationship with an easily accessible health care provider could in itself ease the emotional burden of the disease for persons affected by PD and their care partners.

“I feel she cares. She listens, looks up information, shows me how things work and tells me why she changes medications, for example.” (Study III, Participant 4, Page 1).

### 3.2. Deductive Analysis: Applying the Results on Orem’s Self-Care Deficit Theory

The inductive analysis clearly showed that the social context of people close to the person affected by PD was most important for their ability to handle the disease in everyday life; even though participants also described the importance of support of healthcare professionals to gain new knowledge and develop self-care strategies to manage the impact of the disease. Providing self-management support to patients with long-term conditions has been identified as one of the main tasks of nursing care, the results of the first step of the inductive analysis presented above was applied to a general nursing theory focusing on self-care. This was done to bring together theory and practical reality and to serve as a guide for nurses in their work providing self-management support. Dorothea Orem’s grand nursing theory, The Self-care Deficit theory, was found to be suitable as a theoretical basis. The theory clearly specifies the aim of all nursing care is to help and support patients to develop the abilities needed to manage self-care independently in order to promote and preserve life, health and well-being.

**Figure 2:**
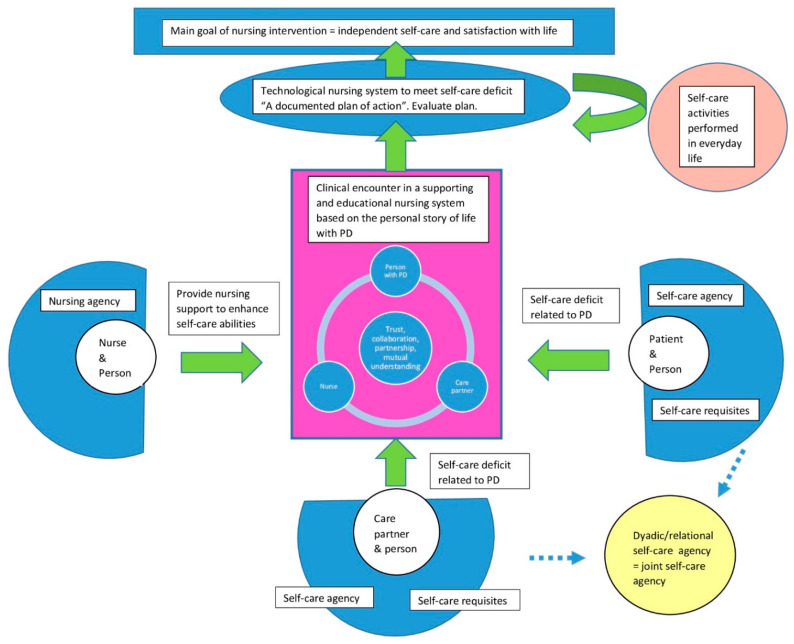
Model of self-management support for persons with long-term conditions in the clinical nursing encounter.

The deductive application of the results to the Self-care deficit theory produced a model of the interactions in a clinical encounter between nurse, person with PD and care partner presented and described below (Figure 2 Self-management support nursing model).

This model acknowledges every encounter between nurse, person with PD and care partner as being a unique encounter. It is a complex encounter between three unique persons. Every encounter will differ from another due to the characteristics that each person brings to the encounter, i.e., personality, previous experiences, emotional state, educational level, ability to relate to other persons, the specific needs and wishes of the participants. Nurses’ profession-specific skills, i.e., factual knowledge of disease/medication, experience in consultation and patient education, ability to understand other person’s emotions—influence their ability to provide nursing support. Orem calls this nursing agency. The encounter will always have its starting point in the personal narrative of what life is like with PD as it is experienced by the person with PD and the care partner. This personal story is the foundation for a mutual understanding of the situation and a prerequisite for the contents and collaboration between nurse, person with PD and care partners in the encounter. The intra-relational encounter between nurse, patient with PD and care partner is represented by the purple box in the middle of the model. All interactions within the encounter take place here. Although every encounter is unique, there are still some general assumptions that can be applied more broadly to describe the nature and structure of the care encounters.

According to Orem, an encounter in a health care setting always includes the meeting of three unique persons but in their situation-specific roles as nurse, patient and care partner. The three persons all have their specific reasons for entering into the encounter. In the model, this is presented by the green arrows pointing from each person into the encounter. The nurse’s aim is to provide nursing care to support self-management abilities, the patient and care partners seek information and advice on how to manage self-care demands brought on by the disease. All persons need to perform activities of self-care in many areas of life to maintain life and health. This is considered a person’s self-care agency, and it is a learned ability developed gradually during life. When being affected by long-term disease, this brings new demands, self-care requisites, that the person needs to recognize and learn how to manage. For persons affected by a progressing disease like PD, the support of a care partner is of great importance. For many, the self-care activities of daily life are seen as a joint concern particularly for activities that might be difficult to perform independently with advanced disease. These were often taken over by the care partner. The care partner can compensate and fulfil the self-care requisite brought on by PD if the person affected by disease can no longer manage it by themselves. The nurse should consider and evaluate this joint or composite self-care ability in every encounter as it is crucial for many patients. In the model, joint self-care ability is represented by the yellow circle in the right corner.

In the encounter between nurse, person with PD and care partner the new requisites brought on by PD are explored and discussed. They include a learning process of how the symptoms of disease can present themselves, as well as learning strategies on how to manage them. The type of intervention to support self-care ability performed for persons with PD primarily takes place in outpatient care and is focused on providing information and strategies on how to handle situations and symptoms occurring in everyday life due to PD. According to the terminology used by Orem, this type of self-management support is called the supporting and educational nursing system. The goal of the encounter is to form a mutual understanding of the situation and together make a plan of action on how to manage the symptoms of disease in everyday life. In the model, this plan of action is represented by the orange circle in the right-hand corner. The plan should be documented by the nurse, and tested by the person affected with PD and care partner in their everyday lives. It is the nurse’s responsibility to evaluate the outcomes of the action plan. If the plan was successful the overall goal of the nursing intervention was met, by providing the support needed for patient and care partners to be independent in performing the self-care actions needed to address the impact of PD. If the plan did not work as intended to resolve the self-care deficit in the situation, nursing intervention is still needed to address the situation. A new encounter between nurse, person with PD and care partner should take place and a new plan of action should be negotiated and again tested in everyday life to address the self-care requisite brought on by PD.

**Figure 3:**
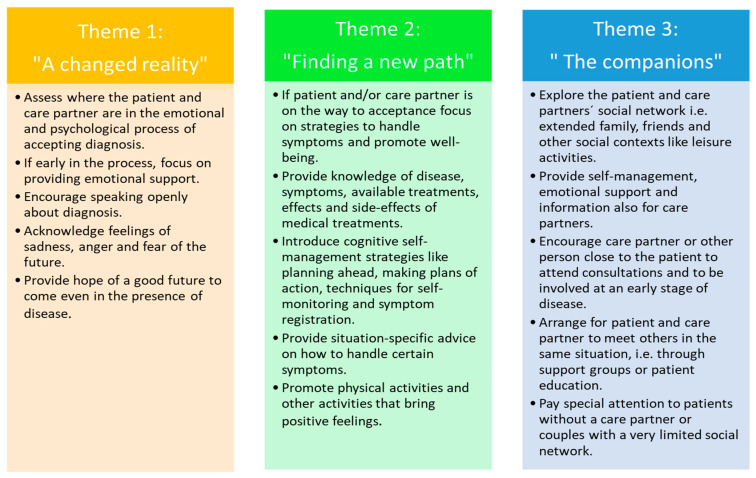
Nursing interventions connected to the themes.

The results of the first step of inductive analysis, described in the three themes above, will be incorporated as new knowledge to improve the nursing agency. The information about the process of acceptance and factors influencing self-management described by the participants will guide the nursing actions in knowing what is important to incorporate in the support to persons with PD and care partners in the clinical encounter (see Figure 3).

During consultation, the nurse should first evaluate where persons with PD and their care partners are in the emotional and psychological process towards accepting diagnosis. If, through the personal narrative being conveyed in the clinical encounter, the nurse understands that the patient and/or care partner is still in the first stage of emotionally reacting to receiving notice of PD, the support needs to focus on providing emotional support. The aim is to encourage speaking openly about the diagnosis and the feelings surrounding it, and to acknowledge the strong feelings of sadness and anger and justify that it is okay to express feelings of unfairness and a fear of the future. The nurse should also focus on providing hope that there is a good future to come and that it is possible to live a fulfilling life even in the presence of PD.

If through the personal narrative the nurse feels that the person with PD and/or care partner has accepted or at least acknowledged the presence of PD in their life and is receptive and looking for ways and suggestions on how to handle their new situation, the support should be directed towards providing strategies to enhance self-management. The nursing consultation can include both situation-specific strategies, addressing how to deal with certain symptoms occurring due to PD and more general strategies used to enhance their overall ability to manage life with PD. These general strategies can include providing medical knowledge of disease including symptoms, available treatment and effects/side effects of medications. Self-management support should also introduce cognitive strategies like planning ahead, making plans of action to handle difficult situations, self-monitoring and registration of health status and symptoms.

As support from other people close to the person with PD is important, nursing assessment should also include exploring the social network of the patient and caregiver including extended family, close friends, other social contexts, i.e., through leisure activities. Nursing support should always include support also for the care partner if there is one, and special consideration should be given to patients without a care partner or with a small social network.

## 4. Discussion

The aim of this study was two-fold. Firstly, it explored self-management and approaches that can be helpful in the process towards independence in self-management of PD in everyday life as experienced by participants of the patient educational intervention NPS. Secondly, it further investigated how the new findings could be understood in relation to Orem’s self-care deficit theory and be applied in the clinical encounter between nurse, patient and care partner.

Three distinct themes were identified describing the process towards self-management of PD in everyday life. The first theme describes the life altering experience of being diagnosed with a long-term condition and the strong emotional reactions often surrounding diagnosis. This event was considered the starting point of a new chapter in life and also the starting point towards self-management of the disease. This initial phase of strong emotional reactions following diagnosis has also been described in other groups of patients [36,37] and should be recognized by nurses working to support persons with long-term disorders. This phase can often be described in terms of a personal crisis and a shock for both the person receiving diagnosis and the persons close to them. During crisis, the susceptibility to new information is often reduced and support should mainly address the strong emotional reactions [12,37].

With the gradual acceptance of the disease, participants felt a need to acquire new knowledge to be able to handle the new circumstances brought on by PD. The strategies articulated by persons with PD and their care partners in this study also show a large overlap with the strategies used by persons with other long-term conditions [38,39]. The support of social networks, peers living in the same situation and support from family for persons living with long-term conditions have been described as essential, especially for children and persons with significant physical or cognitive symptoms [40,41]. This means that in order to support the person with the disease, nursing support must also include support for care partners and other persons close by.

Participants in this study described thinking difficult situations through and making action-plans helpful to manage disease and maintain a sense of control. They also expressed a wish for guidance from healthcare in to grasp which signs could indicate progression of disease. The opportunity to discuss self-management strategies and to discuss the future including possible scenarios ahead with healthcare professionals has previously been identified as unmet needs by PD patients and care partners [42]. Nursing support for persons with PD and their care partners should provide opportunities to discuss future and offer support in making and evaluating action plans to promote independent self-care. A recent study proposed how this could be achieved in clinical care by introducing the concept of a “road-map” allowing patients and care partners the opportunity to discuss this from their personal wishes and needs [43].

The inductive results presented within the context of the three themes above provide important first-hand knowledge of what persons with PD and care partners experience as important factors towards successful management of the disease. This information should be considered and incorporated into practice when providing nursing support for self-management. In a recent review of published qualitative studies investigating self-management components expressed by persons with PD also recognized knowledge and information, self-monitoring strategies, psychological strategies, social engagement, physical exercise and as factors important for self-management [44]. These components were also expressed by persons with PD and care partners in the current study.

Nurses working in clinical care to support patients with long-term disorders sometimes feel unprepared and lacking in adequate training to provide self-management support [5,45]. This study provides a framework that is based on Orem’s grand nursing theory about self-care to guide nurses working in outpatient care and to enhance their understanding of self-management support in the clinical encounter. Recognizing the overall goal of nursing care as helping persons to gain independence in self-care activities brought on by disease can also help nurses to understand their unique role and contribution to patient care. This study is the first to investigate, in depth, how the PD nurse’s self-management support, including the content of nursing actions and interactions with patient and care partners are carried out in the clinical encounter.

The examples in this study are retrieved from persons affected by PD and their care-partners, but it might be reasonable to believe that there are many similarities in the process towards independent self-management also present in other groups of patients affected by long-term conditions. Future studies are needed to explore this. Future studies are also needed to test the applicability of the nursing self-management support model in clinical care encounters presented in this paper also with other groups of patients.

### Strengths and Limitations

This study is important for nurses working in clinical care as it presents a way of thinking about self-management support and provides a model of self-care support that can be applied in the clinical encounter. The model presented is based on a well-known nursing theory, but is still directly applicable in clinical care. This study can also help nurses to understand better their unique contribution to care for persons with long-term conditions.

The data used to explore self-management from the patients’ and care partners’ views used in this study consisted of the three studies exploring a self-management intervention, the Swedish national Parkinson school (NPS). The NPS is a dyadic intervention performed in a small group setting. Although the total number of participants in the three studies included 127 persons with PD and 75 care partners, most of the findings representing the participants’ voices are derived from the two qualitative studies (study I and III) including merely 35 people with PD and 20 care partners. As study II is using self-reported questionnaires with standardized questions and alternatives for answers, and as it was analysed with quantitative methods, there are very limited findings of individual voices in this study. However, even if the individual voices were not apparent in this quantitative study, there were important findings in two domains of one scale showing improved knowledge of self-management strategies, as well as a shift in mind-set to not let the disease control their life, following the NPS self-management support intervention.

Qualitative thematic analysis was used as a method of analysing data in this study. This method is suitable and has previously been used to analyse many types of data and allow for synthesis of different types of data sources and data. It has also been found suitable for and used to inform practice in clinical care [31]. Even though the method allow synthesis of both qualitative and quantitative data, it was obvious during the analysis that the qualitative data were heavily affecting the contents of the themes for the reasons explained above. The analysis was performed by the author of this study who has previous experience with using this method. The author also works part-time as a clinical nurse and therefore has a previous understanding of meeting persons with PD and care partners in clinical consultations. This could have influenced the analysis, for example, in interpreting data and forming themes. The advantage of this pre-understanding is that the author knows what can be of use to other nurses in clinical practice. The model of self-management support presented in this study was presented to other nurses from other hospitals and clinics working to support persons with PD and care partners to collect their views on its value in guiding clinical care and the process. Based on their evaluations, a joint care-plan was made, and evaluating it was further clarified in the model as a result of their input.

The limitations and risk of researcher bias presented above should be taken into account when reading the results of this study. Nevertheless, this study is a valuable contribution to the research literature, and the results can be easily accessed by nurses to improve their abilities to provide self-management support to persons with long-term illness and their care partners. The general model of self-management support in the clinical encounter presented in this paper could be applied to patients affected by various types of long-term conditions, and is not restricted to persons with PD.

## 5. Conclusions

Nursing care for individuals affected by long-term conditions was found to be a unique aspect of their care. The overall goal of nursing care is to provide the information and strategies needed to manage the impact of disease independently in everyday life. This paper contributes to understanding how self-management support can be performed in clinical care. The nursing model, presented in this paper, is based upon Dorothea Orem’s theory of nursing, and describes the interactions between nurse, patient and care partner taking part in the interpersonal meeting. The model can guide nursing interventions and serve as a way of thinking about self-management support in the clinical nursing encounter. In this paper, the model is presented using examples of nursing interventions retrieved from data of persons with PD and their care partners, but the model of self-care support in the nursing encounter can also be useful for nurses supporting persons affected by other long-term conditions.

## Figures and Tables

**Table 1:** Summary of design and participants for studies included in analysis.

Study	Design Data Collection Analysis	Number of Participants (PwPD/CP)	Study Location	Age Range in Years (Median)	Gender (n) Male/Female	Years Since Diagnosis (Median)	Married or Living Together n (%)	Educational Level (n)
**I**	Qualitative group discussions. Audio recordings. Inductive qualitative thematic analysis. Data collected Aug. 2015- June 2016	42 (25/17)	Five outpatient clinics in different parts of Sweden. (3 county and two university hospitals)	68–73 (71)	PwPD: 11/14 CP: 9/8	3–7 years (4.5)	41 (98%)	PS: 13 HS: 12 UD: 17
**II**	Quantitative quasi- experimental case–control study in clinical care. Self-reported questionnaires before/after NPS intervention or 7 weeks’ standard care. Descriptive statistical analysis within and between groups. Data collected Jan 2015- April 2017.	**Total:** 147 (92/55) **Intervention Group**: 78 (48/30) **Control Group**: 69 (44/25)	Five outpatient clinics in different parts of Sweden. (3 county and two university hospitals)	**Intervention Group:** 65–75 (71)	19/29	2–7 years (5)	52 (88%)	PS: 15 HS: 19 UD:25
				CP 68–77 (72)	19/11	**-----**	34 (97%)	PS: 8 HS: 18 UD: 22
				**Control Group:** PwPD 64–75 (68)	30/14	3–8 years (7)	41 (85%)	PS: 12 HS: 8 UD: 15
				CP 67–74 (69)	7/18	**-----**	28 (97%)	PS: 5 HS: 7 UD: 16
**III**	Qualitative observational study with follow-up interviews. Audio recordings and fieldnotes. Inductive constant comparative analysis. Data collected April 2016-Jan 2018.	13 (10/3)	One Swedish university hospital	68–79 (75.5)	PwPD: 5/5 CP: 1/2	2–7 years (4.5)	10 (77%)	PS: 4 HS: 6 UD:3

PwPD: Persons with PD, CP: Care partners, PS: Primary school, HS: High school, UD: University degree. **Study I**: Hellqvist C, Dizdar N, Hagell P, Berterö C, Sund-Levander M. Improving self-management for persons with Parkinson’s disease through education focusing on management of daily life: Patients’ and relatives’ experience of the Swedish National Parkinson School. J Clin Nurs. 2018 Oct;27(19–20):3719–3728. doi:10.1111/jocn.14522. Epub 2018 Jul 30. PMID: 29782061. **Study II**: Hellqvist C, Berterö C, Dizdar N, Sund-Levander M, Hagell P. Self-Management Education for Persons with Parkinson’s Disease and Their Care Partners: A Quasi-Experimental Case-Control Study in Clinical Practice. Parkinsons Dis. 2020 Apr 30;2020:6920943. doi:10.1155/2020/6920943. PMID: 32399171; PMCID: PMC7210533. **Study III**: Hellqvist C, Berterö C, Hagell P, Dizdar N, Sund-Levander M. Effects of self-management education for persons with Parkinson’s disease and their care partners: A qualitative observational study in clinical care. Nurs Health Sci. 2020 Sep;22(3):741–748. doi:10.1111/nhs.12721. Epub 2020 Apr 28. PMID: 32270898.

## Data Availability

The data presented in this study are available on request from the corresponding author. The data are not publicly available due to privacy of participants.

## Supplementary Materials

The following are available online at https://www.mdpi.com/1660-4601/18/5/2223/s1, Supporting information 1: Swedish National Parkinson School.
