# Supplementary material for: Promoting Self-Care in Nursing Encounters with Persons Affected by Long-Term Conditions—A Proposed Model to Guide Clinical Care
Source: Int J Environ Res Public Health. 2021 Feb 24;18(5):2223. doi: 10.3390/ijerph18052223 (PMC7956321; doi:10.3390/ijerph18052223)
Supplement: Supplementary file 1 [file ijerph-18-02223-s001.pdf]

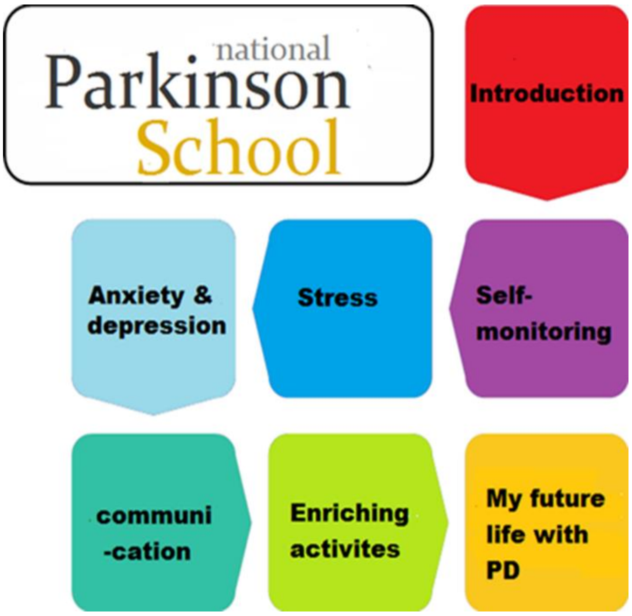

The Swedish National Parkinson School (NPS) is a self-management programme for persons with Parkinson´s disease and their care partners. The main goal of the NPS is to provide the knowledge and tools needed to handle everyday life and enhance life satisfaction. The programme is provided in groups of 12-15 participants. It is offered as a dyadic intervention and care partners are encouraged to attend.

The programme is based on principles of cognitive behavioural therapy. By introducing techniques of self-monitoring and self-observation, participants are introduced to cognitive tools that might be useful to initiate life changes. Promoting awareness of own thoughts, feelings and actions in relation to the impact of PD and replacing negative thoughts with positive and constructive thoughts help to manage difficulties and facilitate an optimistic outlook of the future.

The NPS does not focus on the disease itself, but on how to live a good and fulfilling life and how to reduce the impact of PD in everyday life. The program is based on the idea that the participants first need knowledge about the disease itself, including symptoms and treatment, to understand how it can affect their lives. Self-awareness can help patients and care partners to manage challenges and adjust in order to minimize the disruption of PD in their lives. How people with PD and their families choose to relate to the disease and the changing life situation greatly affects their ability to maintain a good quality of life, despite the difficulties.

NPS consists of seven two-hour sessions where persons with PD and care partners meet in a small group with a certified educator. Each session focuses on a specific topic and consists of an introduction of the topic followed by group discussions where participants share their own thoughts and experiences related to the topic. At the end of the session, participants are instructed to apply experiences from the session to their own lives through practical exercises and home assignments, which are discussed at the beginning of next session. Each session ends with a 15-minute relaxation exercise as a means to manage stress and difficult emotions. In addition to providing knowledge, the NPS also gives participants the opportunity to interact socially and receive peer-support.

The seven themes of the NPS are presented in the figure above and summarized below.

Session 1, Introduction: Participants and the NPS educator introduce themselves sharing some personal background stories and their expectations of the NPS programme. After the presentations, the session focuses on the medical and pathophysiological aspects of PD.

Session 2, Self-monitoring: Introduction of techniques for self-observation, i.e., observing symptoms in relation to the intake of medication and how documenting such observations for some days might lead to better self-awareness.

Session 3, Stress: Participants learn about positive and negative stress and how it affects thoughts and reactions. They are introduced to strategies to “plan ahead” and think stressful situations through in order to find a strategy to deal with them in the future.

Session 4, Depression and anxiety: Dealing with difficult emotions that can arise in everyday life when living with PD. Discussions of when worries become anxiety and down-heartedness turn into depression, and when medical help might be needed.

Session 5, Communication: PD specific issues that might affect communication and lead to misunderstandings, e.g., changes in facial expressions, tone of voice, and difficulties finding words. Strategies to communicate PD related symptoms and needs in clinical care encounters are also discussed.

Session 6, Enriching activities: Focus on what makes participants happy. Examples of activities that are experienced as joyful and that promote feelings of happiness. How to find alternative activities or strategies to maintain these activities even if adjustments to the disease are needed.

Session 7, My life with PD: Summarizes the NPS programme with a brief repetition of what has been discussed and learned. Participants are encouraged to share their thoughts on the programme and what they have found helpful and may be using themselves in everyday life also after conclusion of the programme.
